# Supplementary material for: Psychometric properties of the Mexican version of the opening minds stigma scale for health care providers (OMS-HC)
Source: PeerJ. 2023 Nov 14;11:e16375. doi: 10.7717/peerj.16375 (PMC10655721; doi:10.7717/peerj.16375)
Supplement: Supplemental Information 1 [file peerj-11-16375-s001.pdf]

Escala "Abriendo Mentes" para Personal de Salud (OMS-HC-15)

El siguiente cuestionario busca conocer su opinión acerca de una serie de ideas sobre la salud mental. No hay respuestas correctas o incorrectas. Por favor, marque en cada casilla la respuesta que mejor se ajuste a su opinión.

	Completamente en desacuerdo	En desacuerdo	Ni en acuerdo ni en desacuerdo	De acuerdo	Completamente de acuerdo
1. Me siento más cómodo ayudando a una persona que tiene una enfermedad física que a una persona que tiene una enfermedad mental.	☐	☐	☐	☐	☐
2. Si supiera que un compañero está en tratamiento por una enfermedad mental, cómodo(a) trabajando con él.	☐	☐	☐	☐	☐
3. Si estuviera en tratamiento por una enfermedad mental, es poco probable que lo comente con alguno de mis compañeros.	☐	☐	☐	☐	☐
4. Me sentiría incompetente si tuviera una enfermedad mental y no pudiera resolverlo por mí mismo.	☐	☐	☐	☐	☐
5. Si tuviera una enfermedad mental seguramente no buscaría ayuda profesional.	☐	☐	☐	☐	☐
6. Los empleadores deberían contratar a alguien en tratamiento controlado por una enfermedad mental si es la mejor para el trabajo.	☐	☐	☐	☐	☐
7. Si supiera que el médico que me está tratando tiene una enfermedad mental seguiría yendo con él.	☐	☐	☐	☐	☐
8. Les contaría a mis amigos si tuviera una enfermedad mental.	☐	☐	☐	☐	☐
9. Me inquieta estar cerca de personas con enfermedad mental.	☐	☐	☐	☐	☐
10. Es poco lo que se puede hacer para ayudar a las personas con enfermedades mentales.	☐	☐	☐	☐	☐
11. La mayoría de las personas con enfermedad mental no se esmeran por mejorar.	☐	☐	☐	☐	☐
12. Preferiría que una persona con una enfermedad mental no trabajara con niños, aunque estuviera en un adecuado tratamiento.	☐	☐	☐	☐	☐
13. Los profesionales de la salud no tienen por qué ser defensores de las personas con enfermedades mentales.	☐	☐	☐	☐	☐
14. No me molestaría que una persona con una enfermedad mental fuera mi vecino.	☐	☐	☐	☐	☐
15. Me es complicado sentir compasión por una persona con una enfermedad mental.	☐	☐	☐	☐	☐

Modgill G, Patten SB, Knaak S, Kassam A, Szeto AC. Opening minds stigma scale for healthcare providers (OMS-HC): Examination of psychometric properties and responsiveness. *BMC Psychiatry* 2014; 14(1):120. http://www.biomedcentral.com/1471-244X/14/120.
Kassam A, Papish A, Modgill G, Patten S. The development and psychometric properties of a new scale to measure mental illness related stigma by health care providers: The opening minds scale for Health Care Providers (OMS-HC). BMC Psychiatry 2012; 12:62. DOI: 10.1186/1471-244X-12-62.
