# Supplementary material for: Psychometric properties of the Mexican version of the opening minds stigma scale for health care providers (OMS-HC)
Source: PeerJ. 2023 Nov 14;11:e16375. doi: 10.7717/peerj.16375 (PMC10655721; doi:10.7717/peerj.16375)
Supplement: Supplemental Information 2 [file peerj-11-16375-s002.pdf]

Dataset Information

**Dimension**	**Questionnaire**	**Variable (Label)**	**Response Format**	**Information**
Sociodemographic	General Information	Age (Age)	Numeric	Years of life completed.
		Gender (Gen)	1 Female<br>2 Male	Sex/gender identity.
		Discipline (Disc)	1 Medicine<br>2 Nursing<br>3 Clinical psychology<br>4 Others	Academic field of study. "Others" include social work, occupational therapy, and physiotherapy.
		Occupation (Ocup)	Categorical<br>1 Student<br>2 Professional<br>3 Both	Occupation refers to the state or activity in which a person is involved. Some individuals concurrently hold the roles of students and professionals.
		Educational Level (Edul)	1 Technical education<br>2 Bachelor's degree<br>3 Master's degree<br>4 Doctoral degree	Level of education achieved. In this case, it encompasses a range from technical education to postgraduate programs.
		Academic semester (Acs)	1 1°-4° semester<br>2 5°-6° semester<br>3 7°-8° semester<br>4 9°-10° semester<br>5 Social service<br>6 Specialization<br>7 Graduate program<br>8 Only professional	Refers to the specific period within the academic year during which students are currently enrolled and attending classes.

Mental health stigma	Opening Minds Stigma Scale (OMS-HC)	oms1	Likert 5<br>Completely disagree<br>Disagree<br>Neither agree nor disagree<br>Agree<br>Completely agree	Attitudes of healthcare providers toward people with mental illness
		oms9		
		oms10		
		oms11		
		oms13		
		oms15		
		oms3		Disclosure/help-seeking
		oms4		
		oms5		
		oms8		
		oms2		Social Distance
		oms6		
		oms7		
		oms12		
		oms14		
